# Sawdust-Derived Activated Carbon with Hierarchical Pores for High-Performance Symmetric Supercapacitors

**DOI:** 10.3390/nano12050810

**Published:** 2022-02-28

**Authors:** Yan Zhou, Jun Li, Shilin Hu, Gujie Qian, Juanjuan Shi, Shengyun Zhao, Yulin Wang, Chuan Wang, Jiabiao Lian

**Affiliations:** 1 School of Ecology and Resource Engineering, School of Civil Engineering and Architecture, Wuyi University, Wuyishan 354300, China; zhouyan@wuyiu.edu.cn (Y.Z.); hslwyu2021@163.com (S.H.); sjj1998wyu@163.com (J.S.); zhaoshengyun@wuyiu.edu.cn (S.Z.); ylwanghm@163.com (Y.W.); 2 Key Laboratory of Zhenjiang, Institute for Energy Research, Jiangsu University, Zhenjiang 212013, China; lj42485115@163.com; 3 College of Science and Engineering, Flinders University, Bedford Park, SA 5042, Australia; gujie.qian@flinders.edu.au; 4 Institute of Advanced Synthesis, Jiangsu National Synergetic Innovation Center for Advanced Materials, School of Chemistry and Molecular Engineering, Nanjing Tech University, 30 Puzhu South Road, Nanjing 211800, China

**Keywords:** sawdust, activated carbon, H_3_PO_4_ activation, hierarchically porous structure, symmetric supercapacitors

## Abstract

The recyclable utilization of waste biomass is increasingly important for the development of a sustainable society. Here, the sawdust-derived activated carbon (SD-AC) has been prepared via a convenient H_3_PO_4_-based activation method and further trialed as an electrode for use as a high-performance symmetric supercapacitor. The as-prepared SD-AC possesses a hierarchically porous structure with micropores (0.55 nm) and mesopores (2.58 nm), accounting for its high specific surface area of 621 m^2^ g^−1^, with a pore volume of 0.35 cm^3^ g^−1^. Such a hierarchically porous structure can offer a favorable pathway for fast ion penetration and transportation, enhancing its electrochemical performance. As a result, the SD-AC electrode exhibits a maximum specific capacitance of up to 244.1 F g^−1^ at 1.0 A g^−1^, a high rate capability (129.06 F g^−1^ at 20 A g^−1^), and an excellent cycling performance, with 87% retention over 10,000 cycles at 10 A g^−1^. Of particular note is that the SD-AC-based symmetric supercapacitor achieves a maximum energy density of 19.9 Wh kg^−1^ at the power density of 650 W kg^−1^, with a long-term cycle lifespan. This work showcases the recyclable utilization of waste biomass for the preparation of high-value activated carbon for efficient energy storage.

## 1. Introduction

To solve renewable sources depletion problems, the demand for supercapacitors is sharply increasing, especially for hybrid or electric vehicles [1,2]. Supercapacitors possess excellent energy storage properties, including high specific capacity, fast charging time, high power density, long-term cycle lifespan, low-cost, and good safety [3,4,5,6,7]. At present, porous carbon is recognized as a promising electrode candidate because of its low cost, high surface area and volume, excellent conductivity, and physicochemical stability [8]. As typical eco-friendly resources, biomass has become a global hot topic of sustainable chemistry due to its great potential for practical application [9,10]. For example, rice husk, corn husk, coffee grounds, sugarcane, leaves, and coals have been applied as precursors for making porous carbon [10,11,12,13,14,15,16]. A further attempt has been made to develop competitive porous carbon from readily available wood wastes [17]. Considering that the annual production of wood wastes by the forestry industry worldwide is massive, the reuse of the wood wastes may be both environmentally and economically sustainable [18]. In general, a larger specific surface area (SSA) may offer more active sites for electrolyte components, resulting in higher capacitance [19]. Hence, physical and chemical activation methods have been principally and widely applied for pore formation to increase the SSA. In comparison, the chemical activation method is relatively simple and effective.

In this work, a readily available sawdust (wood waste) was utilized as a carbon source to prepare a porous activated carbon (SD-AC) material via a cost-effective route combing carbonization with H_3_PO_4_ activation in one step. Morphological and structural characterizations were systematically conducted to evaluate the physio-chemical properties of the SD-AC. The results indicated that the as-prepared SD-AC had a high specific surface area of 621 m^2^ g^−1^ and a high pore volume of 0.35 cm^3^ g^−1^, with hierarchical pores, which was favorable for its high-performance energy storage. As expected, the SD-AC electrode exhibited a maximum specific capacitance (244.1 F g^−1^ at 1.0 A g^−1^) with an excellent rate (129.06 F g^−1^ at 20 A g^−1^) and cycling performance (87.03% retention over 10,000 cycles at 10 A g^−1^). Notably, the SD-AC//SD-AC symmetric supercapacitor delivered a maximum energy density of 19.9 W h kg^−1^ at the power density of 650 W kg^−1^, with a long cycling lifespan. This work showcases a good utilization of wood waste to produce high-value porous activated carbon for efficient energy storage.

## 2. Materials and Methods

### 2.1. One-Step Synthesis of the SD-AC

**Figure 1:**
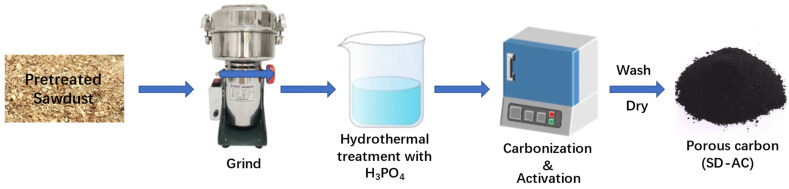
The schematic illustration of the synthetic process for the SD-AC.

Figure 1 shows the schematic illustration of the synthetic process for the SD-AC. First, 10.0 g of Chinese fir sawdust was dispersed in 200 mL of deionized water with magnetic stirring at 600 rpm for 3 h to remove dust and impurities, followed by drying at 80 °C in an oven. Then, the pretreated sawdust was ground (BJ-800A, Baijie Equipment Co., Ltd., Huzhou, China) and screened to be <75 µm. The ground powder was subsequently impregnated into a 85 wt% H_3_PO_4_ solution (the mass ratios of H_3_PO_4_:sawdust was 1:4.8) at room temperature, left for 2 h, and then heated in a furnace to 550 °C for 90 min. The heating rate was 10 °C min^−^^1^. Finally, the furnace was turned off and cooled down to room temperature. The resulting sample was rinsed several times using deionized water (90–100 °C) until the pH of the wash water was near-neutral, and then dried at 105 °C for about 12 h.

### 2.2. Characterizations of SD-AC

Elemental analysis (C, N, H, and O) was performed using a vario EL Cube analyzer (EA, Elementar, Langenselbold, Germany). The surface morphology and microstructure of SD-AC were examined using a JEOL scanning electron microscope (FE-SEM, JSM-7800F, Tokyo, Japan) and a JEOL transmission electron microscope (TEM, JEM-2100F, Japan). For TEM specimen preparation, the SD-AC sample was ultrasonically suspended in ethanol for 5 min, and then several droplets of the above suspension were deposited on a cooper mesh grid. The specific surface area (SSA) and micro- and nano-pores were determined using the N_2_ adsorption method (Tristrar II 3020, Micrometric, Norcross, GA, USA). The SD-AC was also subjected to X-ray powder diffraction analysis (XRD, Bruker D8, Mannheim, Germany) equipped with Cu Kα radiation at 40 kV and 40 mA, with a step width of 0.05° and count time of 0.6 s per step. Raman analysis was performed by the Raman spectrometer with a 532 nm laser for illumination (T6400, Kejie Tianjin, China), taking the 520 cm^−1^ silicon raman band for energy calibration. The surface elemental composition and chemical states were investigated using X-ray photoelectron spectroscopy (XPS, Thermo Scientific ESCALAB 250Xi system with a monochromatic Al Kα X-ray source; C 1s at 284.6 eV for energy calibration). Transmission infrared spectra were collected using a Fourier transform infrared spectrometer (FTIR, Nicolet 20, Thermo Scientific, Waltham, MA, USA). For FTIR specimen preparation, 1–2 mg of the SD-AC was mixed with 200 mg KBr and then and pressed into small discs at 10 tons pressure.

### 2.3. Electrochemical Measurements

A pretreated nickel foam (NF) sheet (1 cm × 1 cm) served as the current collector. The working electrodes were made of 80 wt% SD-AC as the active material, 10% acetylene black as the conductive additive, and 10% poly-tetrafluoroethylene (PTFE) as the binder. The weight of the SD-AC was about 1 mg for each working electrode. The electrolyte was 3 M KOH solution. A platinum plate and an Hg/HgO electrode were used as the counter and reference electrodes, respectively.

The electrochemical analysis of the SD-AC-based electrode was conducted by using an Interface 1000E Gamry electrochemical workstation. Cyclic voltammetry (CV) and galvanostatic charge/discharge (GCD) measurements were performed in a potential window of −1 to 0 V. Electrochemical impedance spectroscopy (EIS) was collected from 0.01 Hz to 100 kHz. The gravimetric specific capacitance *C*_s_ (F g^−^^1^), the energy density (*E*, Wh kg^−1^), and the power density (*P*, W kg^−1^) were determined by Equations (1)–(3), respectively,

(1) $$C_{s}=\frac{I\;\Delta t}{m\;\Delta V}$$

(2) $$E=\frac{1}{3.6}\times \frac{1}{2}C{\left(\Delta V\right)}^{2}$$

(3) $$P=\frac{E}{\Delta t}\times 3600$$

where *I* (A) is the galvanostatic discharge current, Δ*t* (s) is the galvanostatic discharge time, *m* (g) is the net weight of the SD-AC, and Δ*V* (V) represents the potential range.

## 3. Results

### 3.1. Physical Characterization of the SD-AC

The elemental contents of the SD-AC were evaluated by elemental analysis (EA), as summarized in Table 1, indicating that the SD-AC is composed of C, O, H, and N, with the weight percentage of 67.46%, 26.83%, 3.02%, and 0.22%, respectively.

**Figure 2:**
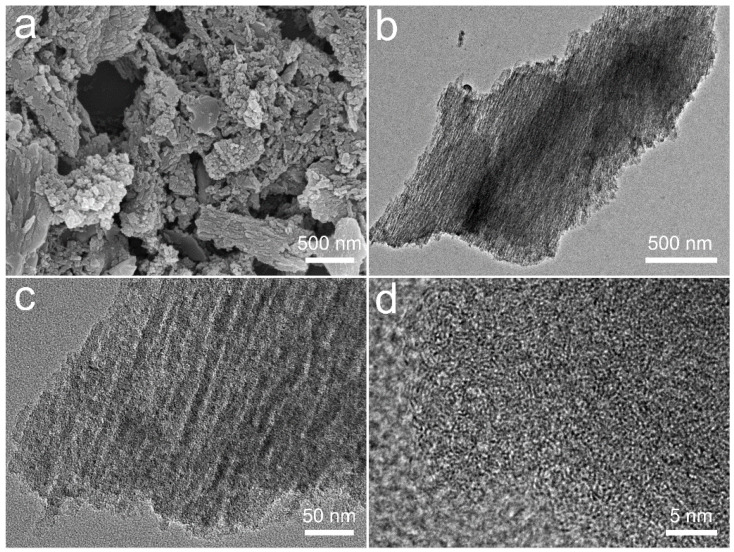
The SEM image (**a**), TEM images (**b**,**c**), and HRTEM image (**d**) of the SD-AC.

The rough and irregular particle is clearly visible under SEM, as observed in Figure 2a. In addition, the external surface of SD-AC is found to be covered with small aligned irregular shaped particles; this is likely caused by pyrolysis at 550 °C, leading to the collapse of the mesopores and the closing of the sub-micron pores during the thermal decomposition of biomass [20]. TEM images (Figure 2b,c) further reveal the unique porous structure of the SD-AC. A high-resolution TEM (HRTEM, Figure 2d) image demonstrates that numerous nanopores exist in the carbon skeletons of the SD-AC, indicating its well-developed porous characteristic. Such a hierarchically porous structure would be highly important for the fast transfer and diffusion of ions.

**Figure 3:**
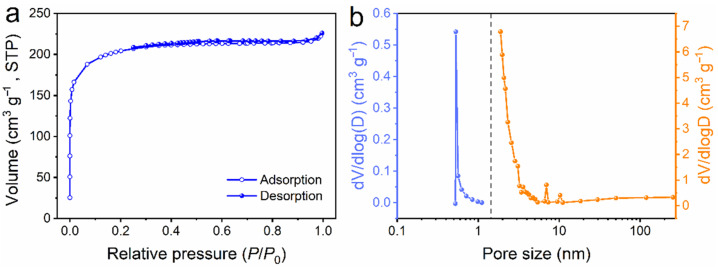
The porosity characteristics of the SD-AC: (**a**) Ar adsorption/desorption plot and (**b**) pore size distribution.

Since the surface area with a porous structure is the key to high-performance electrode materials in energy storage, the Ar adsorption/desorption and pore structure characteristics of SD-AC were further evaluated (Figure 3 and Table 2). As shown in Figure 3a, the isotherm profile is assigned to be type I, with a rapid adsorption of Ar at low relative pressures (*P*/*P*_0_ < 0.03), indicating the presence of a large number of micropores [21]. From *P*/*P*_0_ = 0.03 onwards, the amount of adsorption increases slowly until *P*/*P*_0_ reaches 0.3, suggesting the formation of small-sized mesopores [4]. The size of these micropores and mesopores, derived using the density functional theory (DFT), is measured at about 0.55 and 2.58 nm, respectively (Figure 3b). The BET surface area and pore volume of the SD-AC are calculated to be 621 m^2^ g^−1^ and 0.35 cm^3^ g^−1^, respectively. The high specific surface area with hierarchical pores would provide a favorable pathway for fast ion penetration and transportation [22].

**Figure 4:**
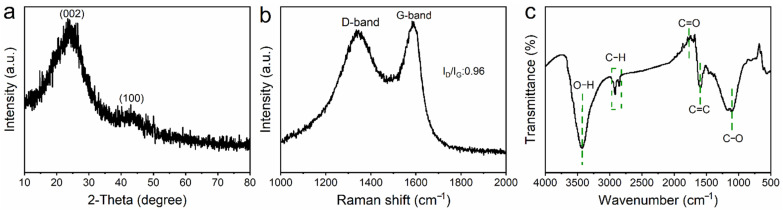
(**a**) The XRD pattern, (**b**) Raman, and (**c**) FTIR spectra of the SD-AC, respectively.

The XRD analysis of the SD-AC (Figure 4a) showed two characteristic broad humps of carbon, including the diffraction from the {002} planes located at about 24.1°, indicative of the disordered structure of carbon [23]. In the Raman spectrum of carbon materials, the G band relates to the graphitic layers (associated with the tangential vibration of the carbon atoms), while the D band is associated with defective graphitic structures, or disordered carbon [24,25]. The intensity ratio of these two peaks partially depends on the graphitization degree [24]. The Raman spectrum of the SD-AC displayed two typical peaks centered at approximately 1335 and 1583 cm^−1^, corresponding to the D and G band of carbon (Figure 4b), respectively. The G band implied the existence of a graphite-like structure in the SD-AC, which can enhance the electrical conductivity of the electrode material [26]. Clearly, the D band intensity of the SD-AC was smaller than its G band intensity, with *I_D_*/*I_G_* = 0.9, suggesting SD-AC is partially graphitized. These carbonaceous materials with partial graphitization are highly suitable for application as electrodes, owing to their high electronic conductivities. The FTIR spectrum (Figure 4c) was recorded to further examine the variations in the functional groups. The bands at 1108 and 1592 cm^−1^ are attributed to C−O (aromatic ether) and C=C aromatic rings, respectively, which are typical for the cellulose and lignin structures, respectively [27]. The band at 1980 cm^−1^ is due to C=O stretching vibrations, while the peaks at 2854 and 2917 cm^−1^ correspond to C−H stretching vibrations. In addition, a strong O–H stretching vibration band centered at 3430 cm^−1^ (typical of phenylic acid) was also observed [28]. These functional groups can accelerate the adsorption of electrolyte ions on the surface of the SD-AC, providing extra faradaic pseudocapacitance [29].

**Figure 5:**
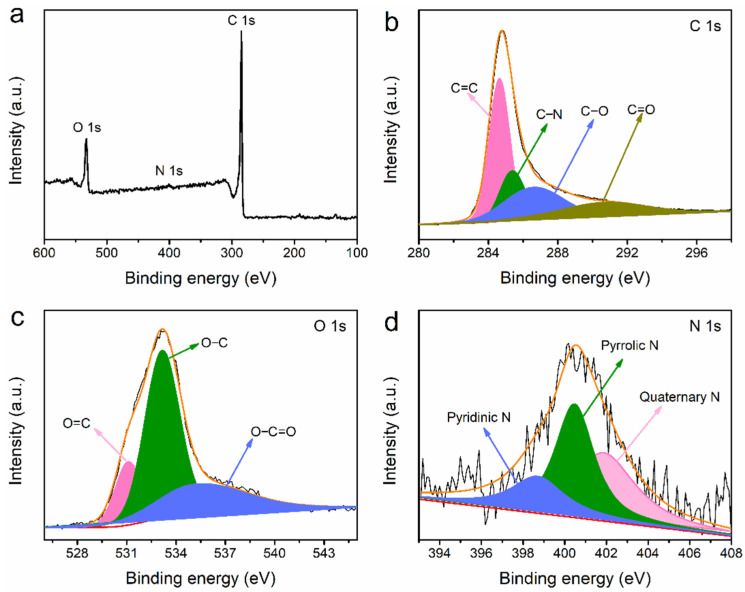
(**a**) The XPS survey, high-resolution; (**b**) C 1s; (**c**) O 1s; (**d**) N 1s spectra of the SD-AC.

The XPS results for SD-AC are presented in Figure 5. As shown in Figure 5a, the SD-AC is dominantly composed of C and O, with minor amounts of N, which is consistent with the EA result. As shown in Figure 5b, the C 1s peak is fitted into four peaks: at 284.7, 285.4, 286.7, and 290.9 eV for C=C, C−N, C−O, and C=O, respectively [29]. It is also found that the peak area of C−N peak is smallest compared to the other peaks, indicating the smallest content of C−N. Figure 5c clearly shows that the O 1s XPS peak is fitted with three peaks at 531.1 eV (O=C), 533.2 eV (O−C), and 535.6 eV (O−C=O). The high-resolution N 1s XPS spectrum (Figure 5d) comprises three components including pyridinic N (398.6 eV), pyrrolic/pyridinic N (400.5 eV), and quaternary N (401.8 eV). The N species at the middle of graphite (quaternary N) is generally less active than other N functionalities [30]. The SD-AC has a small amount of N content (0.22%), but it has been reported to have a positive effect on energy, such as Na^+^ storage [31].

### 3.2. Electrochemical Properties of the SD-AC in a Three-Electrode Test

**Figure 6:**
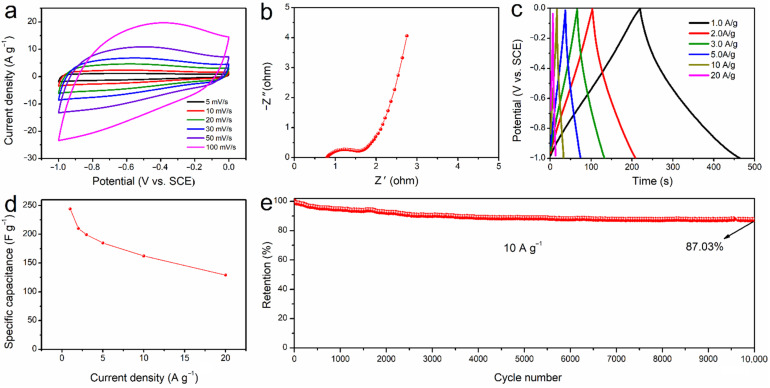
The electrochemical characteristics of the SD-AC electrode in a three-electrode test: (**a**) CV curves at various scan rates of 5–100 mV s^−1^; (**b**) the Nyquist plot; (**c**) the GCD curves at various current densities ranging from 1.0 to 20 A g^−1^; (**d**) specific capacitance as a function of current density; (**e**) cycling performance at 10 A g^−1^ over 10,000 cycles.

The electrochemical characteristics of the SD-AC (Figure 6) was tested in a three-electrode setup in 3 M KOH solution. At various scan rates of 5–100 mV s^−1^, the CV curves clearly show the quasi-rectangle shape of the CV loop in the potential window of −1 to 0 V (Figure 6a), illustrating an excellent electric double layer capacitive behavior and a low contact resistance. The charge transfer characteristics of the SD-AC were investigated using electrochemical impedance spectroscopy (EIS). Figure 6b shows the Nyquist plot of the SD-AC electrode and Figure S1 illustrates the equivalent electrical circuit model used for fitting the Nyquist plot and the values derived from the fitted data. *R*s represents the equivalent series resistance arising from the electrolyte resistance and cell components. The value of *R*s is 0.77 Ω, reflecting the low resistance of the equivalent series. A typical Nyquist diagram includes a depressed semicircle at high frequencies, reflecting the charge-transfer resistance (*R*ct) with a double-layer capacitance CPE (constant phase element). The value of *R*ct can be calculated from the diameter of the semicircle [32,33], which is 0.55 Ω in the present case. At low frequencies, an inclined line at 45° is the phase of Warburg impedance (*Z*_W_) and the nearly vertical straight line is due to the finite length effect [34,35]. Figure 6c displays the GCD curves at different current densities of 1.0 to 20 A g^−1^ with an isosceles quasi-triangular shape, demonstrating the preferential electrical dual-layer storage mechanism [36]. However, the occurrence of faradaic reactions by pseudocapacitance causes the slight deviation from the linear voltage profiles [17]. The specific capacitance values of the SD-AC (Figure 6d) are calculated to be 244.1, 210.2, 199.26, 184.95, 162.2, and 129.06 F g^−1^ at the current densities of 1, 2, 3, 5, 10, and 20 A g^−1^, respectively. Moreover, the SD-AC electrode shows an excellent cycling performance, with 87.03% capacitance over 10,000 cycles at 10 A g^−1^ (Figure 6e). Compared to other biomass-based carbon materials (Table S1), the SD-AC shows a competitive electrochemical performance as a potential candidate for high-performance supercapacitors.

### 3.3. Electrochemical Properties of the SD-AC-Based Symmetric Supercapacitor

**Figure 7:**
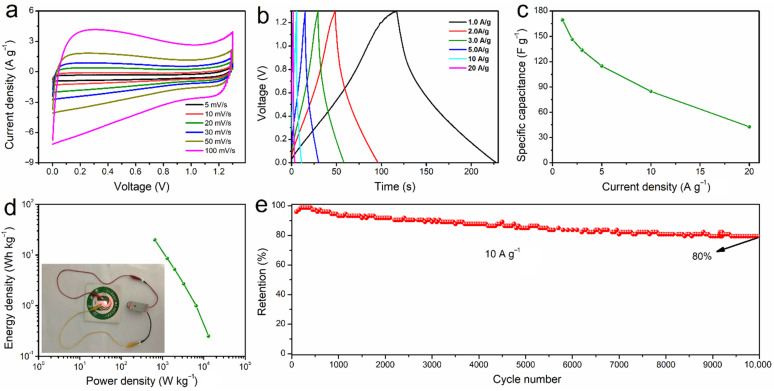
The electrochemical characteristics of the SD-AC//SD-AC symmetric supercapacitors: (**a**) CV curves at various scan rates; (**b**) the GCD curves at various current densities; (**c**) specific capacitance at various current densities; (**d**) the Ragone plot and a photograph of the LED lit by the SD-AC-based symmetric supercapacitors (inset); (**e**) cycling stability at 10 A g^−1^ over 10,000 cycles.

In order to evaluate the SD-AC electrode for practical application, the SD-AC//SD-AC symmetric supercapacitor was assembled and tested in a two-electrode system. Figure 7a illustrates the CV curve profiles obtained at various scan rates of 5–100 mV s^−1^, with typical rectangular-shape, suggesting an excellent reversible capacitive behavior. Humps observation in the CV curves is mainly because of the faradaic process of oxygen-containing functional groups [37]. Figure 7b displays the GCD curves at different current densities. The quasi-symmetrical shape rather than completely symmetrical triangle observed, especially at low current densities, is possibly due to the presence of abundant functional groups. In particular, the O-containing functional groups provide the pseudocapacitive contribution to the total capacitance. The specific capacitance of the device is up to 169.4 F g^−1^ at the current density of 1 A g^−1^ (Figure 7c). The remarkable capacitance of the SD-AC can be attributed to its high specific surface area with hierarchical pores. The Ragone plot in Figure 7d shows the energy/power densities of the SD-AC//SD-AC symmetric supercapacitor, indicating a maximum energy density of 19.9 Wh kg^−1^ at the power density of 650 W kg^−1^ (1 A g^−1^). The SD-AC//SD-AC device can successfully light a red LED (inset in Figure 7d). In addition, the long-term cycle stability of the device was also evaluated. After 10,000 cycles, the device can still retain about 80.0% of capacitance at a high current density of 10 A g^−1^ (Figure 7e). Furthermore, the comparison of our device with other previously reported supercapacitors is shown in Table S2. It is clearly found that the SD-AC electrode exhibits excellent electrochemical performance due to its large surface area and pore volume, as well as the abundant functional groups. The one-step synthesis of the SD-AC in this work could provide a simple sustainable solution for industrial wood waste in its application for the preparation of a high-performance carbon material for electrochemical energy storage.

## 4. Conclusions

In conclusion, the SD-AC with a hierarchically porous structure has been synthesized using a recyclable wood waste biomass via a carbonization method combining H_3_PO_4_ activation in one step at 550 °C. The SD-AC exhibits a high surface area of 621 m^2^ g^−1^ and a very porous structure, with micropores (0.55 nm) and mesopores (2.58 nm). Accordingly, the SD-AC electrode presents the highest specific capacitance of 244.1 F g^−1^ at 1.0 A g^−1^ and a remarkable cycling stability, with 87.03% retention after 10,000 cycles at 10 A g^−1^. Therefore, the SD-AC-based symmetric supercapacitor achieves a high power density of 19.9 Wh kg^−1^ and a power density of 650 W kg^−1^, along with a long cycling lifespan. The excellent electrochemical performance of the SD-AC electrode can be attributed to two main factors: (i) The large surface area and large pore volume with hierarchical pores is beneficial for rapid ion penetration and transportation, boosting its high capacitance and rate capability. (ii) The abundant functional groups provide extra faradaic pseudocapacitance. This work offers a sustainable pathway for utilizing waste biomass to produce high-value activated carbon for efficient energy storage.

## Figures and Tables

**Table 1:** The elemental composition (wt%) of the SD-AC.

Sample	C (%)	O (%)	H (%)	N (%)
SD-AC	67.46	26.83	3.02	0.22

**Table 2:** The parameters of the SD-AC from BET analysis.

Sample	*S*_BET_ (m^2^ g^−1^)	V_total_ (cm^3^ g^−1^)	V_micro_ (cm^3^ g^−1^)	Average Pore Size (nm)
SD-AC	621	0.35	0.24	0.55/2.25

## Data Availability

Not applicable.

## Supplementary Materials

The following supporting information can be downloaded at: https://www.mdpi.com/article/10.3390/nano12050810/s1. Figure S1: The equivalent electrical circuit model used for fitting the Nyquist plot and the values derived from the fitted data. Table S1: Electrochemical performance comparison of biomass-derived carbon materials, Table S2: Electrochemical performance of our SD-AC//SD-AC symmetric supercapacitor in comparison with other previously reported supercapacitors. References [38,39,40,41,42,43,44,45,46,47,48] are cited in the supplementary materials.
