# Supplementary material for: Sawdust-Derived Activated Carbon with Hierarchical Pores for High-Performance Symmetric Supercapacitors
Source: Nanomaterials (Basel). 2022 Feb 28;12(5):810. doi: 10.3390/nano12050810 (PMC8912637; doi:10.3390/nano12050810)
Supplement: Supplementary file 1 [file nanomaterials-12-00810-s001.zip › nanomaterials-1574512 - Supplementary Materials v1.pdf]

nanomaterials

MDPI

Supplementary Materials

# Sawdust-Derived Activated Carbon with Hierarchical Pores for High-Performance Symmetric Supercapacitors

**Yan Zhou [1,†], Jun Li [2,†], Shilin Hu [1], Gujie Qian [3], Juanjuan Shi [1], Shengyun Zhao [1], Yulin Wang [1], Chuan Wang [4,*] and Jiabiao Lian [2,*]**

[1] School of Ecology and Resource Engineering, School of Civil Engineering and Architecture, Wuyi University, Wuyishan 354300, China; zhouyan@wuyiu.edu.cn (Y.Z.); hslwyu2021@163.com (S.H.); sjj1998wyu@163.com (J.S.); zhaoshengyun@wuyiu.edu.cn (S.Z.); ylwanghm@163.com (Y.W.);
[2] Key Laboratory of Zhenjiang, Institute for Energy Research, Jiangsu University, Zhenjiang 212013, China; lj42485115@163.com
[3] College of Science and Engineering, Flinders University, Bedford Park, SA 5042, Australia; gujie.qian@flinders.edu.au
[4] Institute of Advanced Synthesis, Jiangsu National Synergetic Innovation Center for Advanced Materials, School of Chemistry and Molecular Engineering, Nanjing Tech University, 30 Puzhu South Road, Nanjing 211800, China
† These authors contributed equally to this work.
* Correspondence: ias_cwang@njtech.edu.cn (C.W.); jblian@ujs.edu.cn (J.L.)

**Supplementary Materials:**

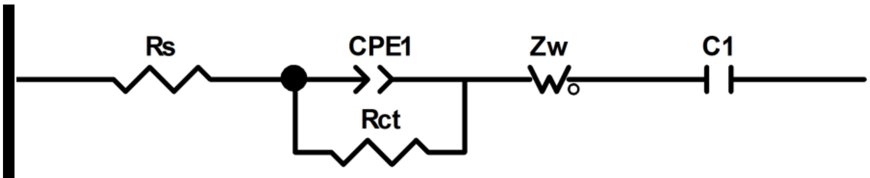


| Element | Freedom | Value | Error | Error % |
|---|---|---|---|---|
| Rs | Free(+) | 0.7743 | 1.0747E-14 | 1.388E-12 |
| CPE1-T | Free(+) | 0.0020049 | 2.9133E-16 | 1.4531E-11 |
| CPE1-P | Free(+) | 0.81143 | 2.0844E-14 | 2.5688E-12 |
| Rct | Free(+) | 0.54626 | 2.937E-14 | 5.3766E-12 |
| Zw-R | Free(+) | 1.794 | 9.9608E-14 | 5.5523E-12 |
| Zw-T | Free(+) | 0.58626 | 1.0815E-13 | 1.8447E-11 |
| Zw-P | Free(+) | 0.32254 | 2.7201E-14 | 8.4334E-12 |
| C1 | Free(+) | 0.18722 | 1.2915E-14 | 6.8983E-12 |

**Figure S1.** The equivalent electrical circuit model used for fitting the Nyquist plot and the values derived from the fitted data.

**Table S1.** Electrochemical performance comparison of biomass-derived carbon materials.

| Reference | Precursor | Electrolyte | Specific Capacitance (F $g^{-1}$) | Cycling performance |
|---|---|---|---|---|
| [1] | Rice straw | 1M $H_2SO_4$ | 332@0.5A $g^{-1}$ | 99% over 5000 cycles @10A $g^{-1}$ |
| [2] | Pine cone | 1M $NaSO_4$ | 137@0.5A $g^{-1}$ | 100% over 10,000 cycles@5.0A $g^{-1}$ |
| [3] | Pomelo peel | 1M $NaNO_3$ | 43.5@1.0A $g^{-1}$ | 90% over 1100 cycles@2.0A $g^{-1}$ |
| [4] | Lotus leaf | 6 M KOH | 298@20A $g^{-1}$ | 90% over 5000 cycles |
| [5] | Pitch | 6 M KOH | 157@100A $g^{-1}$ | - |
| [6] | Macadamia Nut Shell | 1 M $Na_2SO_4$ | 231@1.0A $g^{-1}$ | 87% over 1000 cycles@10A $g^{-1}$ |
| [7] | Coconut shells | $K_2CO_3$ | 91.2@0.2A $g^{-1}$ | 85% over 5000 cycles@0.1A $g^{-1}$ |
| [8] | Cotton stalk | 1 M KOH | 254@0.2A $g^{-1}$ | 96% over 10,000 cycles@1.0A $g^{-1}$ |
| [9] | Rose | 6 M KOH | 208@0.5A $g^{-1}$ | 99% over 25,000 cycles@10A $g^{-1}$ |
| [10] | Rice husk | 6 M KOH | 110.2@0.2A $g^{-1}$ | 78% over 5000 cycles@0.5 A $g^{-1}$ |
| [11] | Mulberry wood | 2 M KOH | 96@5.0 mA $cm^{-1}$ | 87% over 5000 cycles@5.0 mA $cm^{-1}$ |
| [12] | Bagasse | 6 M KOH | 320@0.5A $g^{-1}$ | 93% over 15,000 cycles@10A $g^{-1}$ |
| **This work (SD-AC)** | **Chinese fir sawdust** | **3 M KOH** | **244.1@1.0A $g^{-1}$ 129.1@20A $g^{-1}$** | **87% over 10,000 cycles@10A $g^{-1}$** |

**Table S2.** Electrochemical performance of our SD-AC//SD-AC symmetric supercapacitor in comparison with other previously reported supercapacitors.

| Reference | Source of Electrode Materials | Energy Density (Wh $kg^{-1}$) | Power Density (W $kg^{-1}$) | Cycling performance |
|---|---|---|---|---|
| [1] | Rice straw | 17.4 | 174 | 78% over 5000 cycles@0.5A $g^{-1}$ |
| [2] | Pine cone | 19.0 | 100 | 100% over 10,000 cycles@5A $g^{-1}$ |
| [3] | Pomelo peel | 17.1 | 420 | - |
| [4] | Lotus leaf | 9.2 | 491 | 90% over 5000 cycles@5A $g^{-1}$ |
| [5] | Pitch | 20 | 100 | - |
| [7] | Coconut shells | 23.1 | 135 | 89.2% over 5000 cycles@2A $g^{-1}$ |
| [8] | Cotton stalk | 18.1 | 450 | - |
| [9] | Rose | 20.3 | 525 | 92% over 10,000 cycles@5A $g^{-1}$ |
| [12] | Bagasse | 20 | 182 | 91% over 10,000 cycles |
| [13] | Soya Bean pods | 22.3 | 450 | 91% over 10,000 cycles@5A $g^{-1}$ |
| **This work** | **Chinese fir sawdust** | **19.9** | **650** | **80% over 10,000 cycles @10A $g^{-1}$** |

## References


1. Sudhan, N.; Subramani, K.; Karnan, M.; Ilayaraja, N.; Sathish, M. Biomass-derived activated porous carbon from rice straw for a high-energy symmetric supercapacitor in aqueous and non-aqueous electrolytes. *Energy Fuels.* **2016**, *31*, 977–985.
2. Bello, A.; Manyala, N.; Barzegar, F.; Khaleed, A.A.; Momodu, D.Y.; Dangbegnon, J.K. Renewable pine cone biomass derived carbon materials for supercapacitor application. *RSC Adv.* **2016**, *6*, 1800–1809.
3. Peng, C.; Lang, J.; Xu, S.; Wang, X. Oxygen-enriched activated carbons from pomelo peel in high energy density supercapacitors. *RSC Adv.* **2014**, *4*, 54662–54667.
4. Qu, S.; Wan, J.; Dai, C.; Jin, T.; Ma, F. Promising as high-performance supercapacitor electrode materials porous carbons derived from biological lotus leaf. *J. Alloys Compd.* **2018**, *751*, 107–116.
5. Guo, Y.; Shi, Z.; Chen, M.; Wang, C. Hierarchical porous carbon derived from sulfonated pitch for electrical double layer capacitors. *J. Power Sources.* **2014**, *252*, 235–243.
6. Yan, X.; Jia, Y.; Zhuang, L.; Zhang, L.; Wang, K.; Yao, X. Defective carbons derived from macadamia nut shell biomass for efficient oxygen reduction and supercapacitors. *ChemElectroChem*. **2018**, *5*, 1874–1879.
7. Xia, J.; Zhang, N.; Chong, S.; Chen, Y.; Sun, C. Three-dimensional porous graphene-like sheets synthesized from biocarbon via low-temperature graphitization for a supercapacitor. *Green Chem.* **2018**, *20*, 694–700.
8. Tian, X.; Ma, H.; Li, Z.; Yan, S.; Ma, L.; Yu, F.; Wang, G.; Guo, X.; Ma, Y.; Wong, C. Flute type micropores activated carbon from cotton stalk for high performance supercapacitors. *J. Power Sources.* **2017**, *359*, 88–96.
9. Zhao, C.; Huang, Y.; Zhao, C.; Shao, X.; Zhu, Z. Rose-derived 3D carbon nanosheets for high cyclability and extended voltage supercapacitors. *Electrochim. Acta*. **2018**, *291*, 287–296.
10. Zhang, W.; Lin, N.; Liu, D.; Xu, J.; Sha, J.; Yin, J.; Lin, H. Direct carbonization of rice husk to prepare porous carbon for supercapacitor applications. *Energy.* **2017**, *128*, 618–625.
11. Ma, Y.; Yin, J.W.; Liang, H.Q.; Yao, D.X.; Xia, Y.F.; Zuo, K.H.; Zeng, Y.P. A two step approach for making super capacitors from waste wood. *J. Cleaner. Prod.* **2021**, *279*, 123–786.
12. Feng, H.; Hu, H.; Dong, H.; Xiao, Y.; Cai, Y.; Lei, B.; Zheng, M. Hierarchical structured carbon derived from bagasse wastes: A simple and efficient synthesis route and its improved electrochemical properties for high-performance supercapacitors. *J. Power Sources.* **2016**, *302*, 164–173.
13. Liu, Z.; Zhu, Z.; Dai, J.; Yan, Y. Waste biomass based-activated carbons derived from soybean Pods as electrode materials for high-performance supercapacitors. *ChemistrySelect*. **2018**, *3*, 5726–5732.
